# Bacterial protein MakA causes suppression of tumour cell proliferation via inhibition of PIP5K1α/Akt signalling

**DOI:** 10.1038/s41419-022-05480-7

**Published:** 2022-12-06

**Authors:** Eric Toh, Palwasha Baryalai, Aftab Nadeem, Kyaw Min Aung, Sa Chen, Karina Persson, Jenny L. Persson, Bernt Eric Uhlin, Sun Nyunt Wai

**Affiliations:** 1 Department of Molecular Biology, Umeå Centre for Microbial Research (UCMR), Umeå University, SE-90187 Umeå, Sweden; 2 The Laboratory for Molecular Infection Medicine Sweden (MIMS), Umeå University, SE-90187 Umeå, Sweden; 3 Department of Chemistry, Umeå University, SE-90187 Umeå, Sweden

**Keywords:** Molecular biology, Cell biology, Cancer

## Abstract

Recently, we demonstrated that a novel bacterial cytotoxin, the protein MakA which is released by *Vibrio cholerae*, is a virulence factor, causing killing of *Caenorhabditis elegans* when the worms are grazing on the bacteria. Studies with mammalian cell cultures in vitro indicated that MakA could affect eukaryotic cell signalling pathways involved in lipid biosynthesis. MakA treatment of colon cancer cells in vitro caused inhibition of growth and loss of cell viability. These findings prompted us to investigate possible signalling pathways that could be targets of the MakA-mediated inhibition of tumour cell proliferation. Initial in vivo studies with MakA producing *V. cholerae* and *C. elegans* suggested that the MakA protein might target the PIP5K1α phospholipid-signalling pathway in the worms. Intriguingly, MakA was then found to inhibit the PIP5K1α lipid-signalling pathway in cancer cells, resulting in a decrease in PIP5K1α and pAkt expression. Further analyses revealed that MakA inhibited cyclin-dependent kinase 1 (CDK1) and induced p27 expression, resulting in G2/M cell cycle arrest. Moreover, MakA induced downregulation of Ki67 and cyclin D1, which led to inhibition of cell proliferation. This is the first report about a bacterial protein that may target signalling involving the cancer cell lipid modulator PIP5K1α in colon cancer cells, implying an anti-cancer effect.

## Introduction

*Vibrio cholerae*, a highly motile Gram-negative bacterium, is the causal agent of the diarrhoeal disease cholera. It is an extracellular facultative human pathogen that alternates between aquatic and intestinal habitats throughout its life cycle [1, 2]. The study of how *V. cholerae* and other *Vibrionaceae* survive and flourish in harsh environments is a major focus for researchers [3].

Recently, we identified a novel *V. cholerae* cytotoxin, MakA (motility associated killing factor A), its secretion was mediated by the flagellum and MakA was shown to function as a potent virulence factor in both zebrafish and *C. elegans*, implying that it functions as an environmental fitness factor for *V. cholerae* against predatory organisms in its natural aquatic habitats [4]. MakA protein also displayed substantial cytotoxicity in vitro to cultured mammalian cells, notably against several tested cancer cell lines, although it seemed to be less harmful to non-transformed cells, prompting it to be considered in studies aimed towards new strategies for developing therapeutic agents against colon cancer [5]. Additionally, structural characterization of MakA [4] revealed similarities to the protein ClyA, a pore-forming toxin first discovered in non-pathogenic *Escherichia coli* [6, 7] and later in *Salmonella enterica* [8]. MakA was also found to be structurally related to other ClyA family pore-forming toxins, including the hemolysin BL binding component B (HBL-B) and the NheA component of the Nhe nonhemolytic enterotoxin from *Bacillus cereus* [4]. Furthermore, we found the *makA* gene in a gene cluster, present in most *V. cholerae*, that encodes four additional proteins: MakB, MakC, MakD, and MakE [4, 9]. Our studies of the Mak proteins from *V. cholerae* revealed that MakA could form a tripartite cytolytic complex together with MakB and MakE, although neither of the three proteins exhibits such cytolytic activity on its own [9]. However, when subjected to low pH conditions in the presence of lipid membranes, the MakA protein seems to adopt a conformation that oligomerises and remodels lipid membranes into tubular protein-lipid structures, leading to loss of cell membrane integrity that ultimately leads to cell death [10].

Lipids, most notably phospholipids, are essential components of all cellular membranes. Phospholipids including phosphatidylethanolamine, phosphatidylglycerol, phosphatidylserine, phosphatidylcholine, phosphoinositides, phosphatidic acid, and sphingomyelin are involved in a wide variety of biological processes [11]. Earlier studies have demonstrated a correlation between an increase in phospholipid content and an increase in cell transformation and tumour growth [12–15]. For example, breast cancer tissue contains more phospholipids than adjacent healthy breast tissue [16–19]. Phosphoinositides (PIs), which include the precursor phosphatidylinositol (PtdIns) and their phosphorylated derivatives, polyphosphoinositides, are the most studied phospholipids (PPIs) in cell biology [20]. PtdIns can be phosphorylated by the lipid kinase PIP5K1α (Phosphatidylinositol-4-phosphate 5-kinase alpha) and PI3K (phosphatidylinositol 3-kinase) at three of the five hydroxyl groups on the inositol ring, yielding different types of PPIs: PtdIns(3,4,5)P3 (PIP3), PtdIns(4,5)P2 (PIP2), PtdIns(3,4)P2 (PIP4), PtdIns4P (PIP) [21]. The PIP5K family of lipid kinases consists of the three isozymes α, β, and γ [22–24]. PIP2 (phosphatidylinositol 4,5-bisphosphate) is generated by the kinase PIP5K1α through phosphylation of PIP, and it then serves as a substrate for the synthesis of PIP3 (phosphatidylinositol 3,4,5-triphosphate) by the action of PI3K. PI3K promotes the Akt signalling pathway via the phospholipids PIP2 and PIP3 [25], with the downstream signalling impact of promoting cell survival and proliferation. PIP5K1α is a predominant kinase to produce phosphatidylinositol 4,5-bisphosphate PI(4,5)P2 (PIP2) for the activation of PI3K/Akt pathways [26, 27]. Overexpression of PIP5K1α promotes tumour growth and invasiveness by increasing the activity of PI3K/Akt in mouse xenograft models [28, 29]. Abnormal expression of AR and PIP5K1α/Akt pathways cooperatively contributes to growth, survival, and invasiveness in various types of metastatic cancer [28, 30]. However, little is known about the potential therapeutic usefulness of lipid kinases as possible targets in cancer.

The cell cycle is a physiological process that entails a series of events that occur during the division of a cell. The cell cycle is divided into two phases: DNA synthesis, S phase, and a mitotic M phase, which are separated by two gap phases (G 1 and G 2) [31]. This process is tightly regulated in order to regulate cell growth, apoptosis, and other critical events for cell survival [32]. Cell cycle progression is regulated through a complex interaction between cyclins, cyclin-dependent kinases (CDKs), and cyclin-dependent kinase inhibitors (CKIs). The binding of CKIs to CDKs controls the CDK activity, promoting or inhibiting cell cycle progression. The CKIs are classified into two main groups: the INK4 (p15, p16, p18, and p19) and CIP/KIP (p21, p27, and p57) [33, 34]. The p27 gene is located on chromosome 12p13 and its protein product can bind to cyclin (A, B, D, and E)/CDK (1, 2, 4, and 6) complexes, altering the conformation of the CDK active site, resulting in disengagement of the cyclin from the CDK [35]. p27 is a tumour suppressor that inhibits cancer cell proliferation by deceptive mechanisms such as proteolytic degradation and nuclear exclusion to promote cell proliferation [36]. The p27 protein has a 44 percent identity with p21 at its amino terminus and, like p21, inhibits cyclin-induced phosphorylation of retinoblastoma protein (Rb). This results in Rb binding to the transcription factor E2F1, inhibiting transcription of cyclins (cyclin A, cyclin D, and cyclin E) required for G1/S transition [37].

The PI3K/Akt signalling pathway is the most essential cellular route involving p27 activity. Downregulation of p27, which triggers cell cycle progression, is linked to the activation of Akt, a PI3K downstream effector [38]. The levels of p27 protein can be used as a prognostic marker in a wide variety of human cancers [39, 40]. Several studies have demonstrated a link between decreased p27 expression and advanced tumour grade and stage in human colorectal, gastric, breast and other cancers [41–44]. Additionally, a substantial link exists between lower p27 protein expression in tumours and decreased survival in individuals with colorectal, gastric, breast, and oesophageal squamous cell carcinoma [45–48].

Several studies have demonstrated that bacterial toxins may affect the phospholipid levels of their hosts. For instance, C3 toxin from *Clostridium botulinum* has been observed to reduce GTPase Rho protein activity and cellular PIP2 levels [49]. *E. coli* CNF1, which activates Rho, Rac, and Cdc42, on the other hand, has been shown to activate a cytoskeleton-associated PI4-P 5-kinase in a time- and dose-dependent manner [50]. In addition, the *Pasteurella multocida* toxin activates Gq, the G12–13 subunits of heterotrimeric G-proteins, by an unidentified mechanism involving the conversion of GDP- to GTP-Gq. GTP-Gq dissociation from the G complex activates PLC1, which catalyzes the hydrolysis of PIP2 into IP3 (inositol triphosphate) and diacylglycerol, leading to Ca2+ mobilization, PKC activation, and downstream signalling [51–53].

In the present study, we evaluated the effects of MakA on the host cell PIP5K1α lipid-signalling pathway and the mechanism by which MakA inhibits cell proliferation and induces cell cycle arrest, particularly in HCT8 colon cancer cells. We found that MakA could inhibit the PIP5K1α/Akt lipid-signalling pathway, resulting in increased expression of the tumour suppressor p27. Furthermore, we demonstrated that the combination of MakA with the PIP5K1α inhibitor ISA-2011B caused a more significant decrease in tumour cell proliferation when compared to HCT8 cells treated with MakA or ISA-2011B alone. Our observations suggest that MakA could be a potential candidate for the development of new therapeutic agents against colorectal cancer by targeting PIP5K1α lipid-signalling.

## Materials and methods

### Bacterial strains

The bacterial strains used in this study were the *Escherichia coli* strains *E. coli* Top10/pBAD18 and *E. coli* Top10/p*makDCBA* [4]. Both strains were grown at 37 °C in LB broth containing 100 µg/mL carbenicillin and gene expression of the plasmids was induced by adding 0.1% L-arabinose. For the *C. elegans* experiments, bacteria were cultured on nematode growth medium (NGM) agar plates containing 100 µg/mL carbenicillin and 0.1% L-arabinose.

### Cell culture

The human colon carcinoma cell lines (HCT8 and DLD1), mouse colon cancer cell line (CT26) were purchased from the American Type Culture Collection (ATCC), Manassas VA. The cells were cultured in Roswell Park Memorial Institute (RPMI) 1640 medium (GIBCO) supplemented with 10% fetal bovine serum (FBS), 1% penicillin/streptomycin, and non-essential amino acids at 37 °C with 5% CO_2_.

### MakA protein purification and labelling

The MakA protein was purified as previously reported [4]. For the immunofluorescence experiments, MakA was labelled with Alexa Fluor 568 using an Alexa Fluor 568 protein labelling kit (Thermo Fisher) according to the manufacturer’s instructions.

### Antibodies

The primary antibodies used in this study include: Actin (#A5441, WB = 1:5000, Sigma Aldrich), Histone H3 (#06755, Western blot (WB) = 1:5000, Millipore), PIP5K1α (#15713-1-AP, WB = 1:5000, Immunofluorescence (IF) = 1:300, Proteintech), phosphoAkt-Ser473 (pAkt Ser473, #4060, WB = 1:2000, IF = 1:200, Cell Signalling), Akt (#9272, WB = 1:1000, Cell Signalling), CDK1 (#610038, WB = 1:1000, BD Transduction Laboratories), CDK2 (#610145, WB = 1:3000, BD Transduction Laboratories), Cyclin D1 (#MA5-14512, WB = 1:500, Invitrogen ThermoFisher Scientific), Cyclin A1 (#556600, WB = 1:100, BD Transduction Laboratories), p27 (#PA5-27188, WB = 1:1000, IF = 1:300, Invitrogen ThermoFisher Scientific), E2F1 (#3742, WB = 1:1000, Cell Signalling), PIP2 (#MA3-500, IF = 1:300), Ki67 (#550609, IF = 1:200). For Western blotting, the secondary antibodies Goat anti-rabbit-HRP (#AS09602, WB = 1:5000) and Rabbit anti-mouse-HRP (#P0260, WB = 1:5000) were purchased from Agrisera and Dako respectively. For immunofluorescence, fluorescently conjugated Alexa-488 secondary antibodies (#A21202, #A21206, 1:300, Invitrogen) were used.

### PIP2 and PIP5K1α immunodetection in *Caenorhabditis elegans* (*C. elegans*)

For immunostaining, the worms fed with *E. coli* Top10/pBAD18 vector or *E. coli* Top10/p*makDCBA* were washed in 1 × PBS (phosphate-buffered saline), fixed in 4% paraformaldehyde, washed with PBST (0.1% TritonX-100 in 1 × PBS), and blocked with 5% normal goat serum (NGS) in PBST. The primary antibody to detect PIP2 was anti-PIP2 mouse antibodies (1:200, Echelon). Fluorescently conjugated Alexa-488 secondary antibody (1:500, Jacksson Immuno-Research) plus phalloidin-Cy3 (1:1000, Jacksson Immuno-Research) and DAPI (1 µg/ml) were used for visualization of detected PIP2. Samples were mounted in Fluoromount-GTM medium (Southern Biotech) and images were taken by a Zeiss ApoTome fluorescence microscope.

For the immunoblot analysis, total protein extraction was prepared from L4 stage worms in 20 µl lysis buffer (50 mM Tris-HCl, pH7.5; 100 mM NaCl; 1 mM EDTA with protease inhibitor). Antisera used in immunoblot analyses were anti-PIP5K1α polyclonal antiserum 1:5000 dilution, anti-actin polyclonal antiserum 1:15000 dilution, and anti-Histone H3 1:1000 antiserum.

### Immunoblot analysis

HCT8, DLD1 and CT26 cancer cells (3 × 10^5^ cells/well) were grown overnight in a 6-well plate (ThermoFisher Scientific), then treated with MakA or ISA-2011B alone for 24 h and 48 h or MakA and ISA-2011B in combination for 48 h. Subsequently, the cells were washed with 1× cold PBS and lysed with ice-cold lysis buffer (20 mM Tris-HCl pH 8.0, 300 mM KCl, 10% Glycerol, 0.25% Nonidet P-40, 0.5 mM EDTA, 0.5 mM EGTA, 1 mM PMSF, 1× complete protease inhibitor (Roche)). The cell lysates were normalized to the same concentration, mixed with 4× sample buffer, and boiled for 10 min. Equal amounts of protein were loaded on a 12% polyacrylamide gel and separated by SDS-PAGE followed by transfer to a nitrocellulose membrane. The membrane was incubated with 5% skimmed milk PBST for blocking at RT for 1 h. After blocking, membranes were incubated with primary antibodies (in 5% skimmed milk) at 4 °C overnight. The next day, the membranes were washed three times for 10 mins with PBST (0.1%) and then incubated with appropriate HRP-conjugated secondary antibodies in blocking buffer (5% skimmed milk, RT, 1 h). The immunoreaction bands were developed using the ECL BIO-RAD Clarity^TM^ Western substrate (Bio-Rad Laboratories, Inc., USA) and images were recorded using LAS-4000 software on a LAS-4000 imager (GE Healthcare, Chalfont St Giles, UK).

### Plasmids and transfection

For transfection, the plasmids pLPS-3’EGFP (empty vector control) and pLPS-PIP5K1α-3’EGFP (carrying full length PIP5K1α cDNA as PIP5K1α-EGFP) were used. The plasmids were introduced into HCT8 colon cancer cells using TransIT-X2 transfection reagent (#MIR 6004) according to the manufacturer’s protocol (Mirus Bio LLC, Madison, WI, USA). Subsequently, transfected cells were used for co-localization experiment of MakA with PIP5K1α as well as for immunostaining of the cell proliferation marker Ki67.

### Flow cytometry

Cell cycle analysis after treatment of HCT8 cells with MakA was investigated by flow cytometry. HCT8 cells (4 × 10^5^ cells) were seeded in a 6-well plate overnight and then treated with MakA or vehicle (20 mM Tris-HCl) for 24 h. After treatment, the cells were harvested and washed (1000 rpm for 5 min) in fresh RPMI medium. Subsequently, the cells were fixed with 70% ice-cold ethanol for 30 min at 4 °C. The cells were then washed twice with PBS, treated with and without 50 µg/ml RNase for 30 min, and stained with 50 µg/ml propidium iodide (PI) for 15 min at room temperature in the dark. This was followed by washing of the cells and resuspension in PBS. The samples were analyzed by flow cytometry using a BD Accuri C6 flow cytometer (BD Bioscience) and the percentage of cells in each cell cycle phase was determined using the BD Accuri C6 software.

### Clonogenic assay

A clonogenic assay was conducted to investigate the impact of MakA on tumour cell proliferation. Briefly, HCT8 cells (5 × 10^3^ cells/well) were seeded overnight in a 24-well plate and treated with MakA (500 nM), ISA-2011B (25 μM) or a combination of MakA (500 nM) and ISA-2011B (25 μM) for 4 days. The cells were then fixed with 4% PFA for 30 min and subsequently stained with 0.5% crystal violet for 30 min at RT. The number of colonies (>50 cells) was examined in duplicate wells.

### Immunofluorescence

HCT8 (3 × 10^4^ cells) or (5 × 10^3^ cells) harbouring pLPS-3’EGFP (empty vector control) and pLPS-PIP5K1α-3’EGFP (PIP5K1α expression clone) were cultured on a coverslip in a 24-well plate or an 18-well ibidi chamber slide overnight. The cells were then treated with either vehicle, Alexa568-MakA (500 nM), MakA (500 nM), ISA-2011B (25 µM), or a combination of MakA (500 nM) and ISA-2011B (25 mM). Subsequently, the cells were fixed with 3.7% paraformaldehyde for 30 min at room temperature (RT) and washed with phosphate buffer saline (PBS). The cells were permeabilized with 0.5% Triton X-100 in PBS for 15 min followed by blocking with 5% foetal bovine serum (FBS) in PBS for 60 min at RT. The cells were washed and then incubated with primary antibodies (PIP5K1α, PIP2, pAkt, p27 and Ki67) overnight at 4 °C. The next day, the cells were washed with PBS and then incubated with their respective secondary Alexa 488-conjugated secondary rabbit or mouse antibodies for 2 h at RT. Subsequently, the cells were washed and the nuclei counterstained with DAPI for 5 min at RT followed by washing with PBS. The samples were mounted on a slide in Fluoromount aqueous mounting medium (#F4680, Sigma). Using an EZC1 Eclipse laser scanning confocal microscope (Nikon) with a 63×/1.4 plan Apo λs lens or a Leica SP8 inverted confocal system (Leica Microsystems) with an HC PL APO 63/1.40 oil immersion lens, the images were obtained. Using the NIS-Elements (Nikon) or LasX (Leica Microsystems) and ImageJ software, images were collected and processed. Using Image J, the fluorescence intensity of PIP5K1α, PIP5K1α-EGFP, and EGFP were measured.

### Statistical analysis

The data are presented as the mean ± s.d. of at least two independent experiments. On GraphPad Prism, a one-way ANOVA and unpaired T-Test were conducted to establish statistical significance among different treatment conditions, whereby *p ≤ 0.05, **p ≤ 0.01, ****p ≤ 0.0001 and ns = non-significant.

## Results

### The effect of bacteria expressing *mak* operon proteins on PIP5K1α lipid signalling in *C. elegans*

**Fig. 1:**
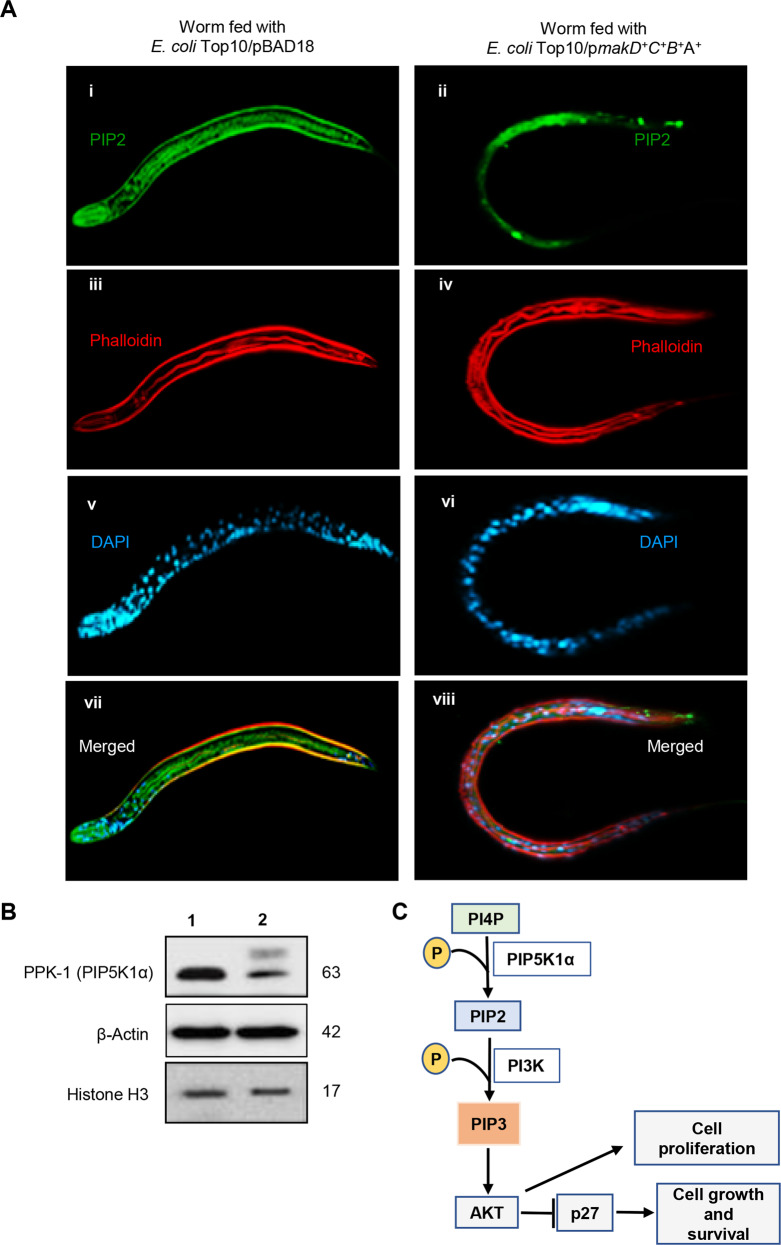
MakA-expressing bacteria inhibit PIP5K1α phospholipid signalling in *C. elegans*. **A** *C. elegans* were fed with *E. coli* Top10/pBAD18 or *E. coli* Top10/*pmakDCBA*. PIP2 levels was assessed by confocal microscopy using anti-PIP2 mouse antibody. Nuclei were counterstained with DAPI (blue) and actin filaments with phalloidin (red). **B** Western blot analysis of levels of PPK-1 (PIP5K1α ortholog) in total cell lysates of worms fed with *E. coli* Top10/pBAD18 or *E. coli* Top10/*pmakDCBA*. **C** Schematic representation of phospholipid signalling in human host cells.

It has been shown that phospholipids located in the membranes of host cells may be targets of bacterial pathogens in modulation of host cell signalling, and it has also been proven that lipids play a significant role in many phases of host-pathogen interactions [54]. In our earlier studies of how the bacterial strains expressing *makDCBA* genes kill *C. elegans*, we examined the morphological changes that occur in worms over time. Several morphological defects were observed in worms feeding on bacteria expressing all four Mak proteins. Specifically, the lipid droplets normally seen in the cytoplasm of intestinal cells were greatly decreased in worms feeding on MakA-expressing bacterial strains [4]. To gain insight into if bacterial strains harbouring the *makDCBA* genes may influence lipid signalling in *C. elegans*, we examined the expression of PIP2 and PPK-1, an ortholog of PIP5K1α in *C. elegans*, in worms exposed to the *E. coli* strain expressing all four proteins encoded by the *makDCBA* operon (*E. coli* Top10/p*makDCBA*) or *E. coli* Top10/pBAD18 as a control. We compared the levels of PIP2 in the worms by immunofluorescence microscopy analysis using PIP2 antiserum as described in the materials and methods. As shown in Fig. 1A, the PIP2 level was reduced in the worm fed with *E. coli* expressing the *mak* operon in comparison with the worm fed with *E. coli* harbouring the vector control. Furthermore, immunoblot analysis of cell lysates derived from these L4 stage worm samples revealed a substantial reduction in the PIP5K1α level in worms exposed to *E. coli* Top10/p*makDCBA* (Fig. 1B). Taken together, these data suggest that Mak proteins may target the PIP5K1α phospholipid-signalling pathway in *C. elegans*, and the results prompted us to investigate if the corresponding pathway in mammalian cells would be similarly affected.

### MakA co-localizes with PIP5K1α at the cell membrane of HCT8 cells and reduces the activity of PIP5K1α

PIP5K1α phosphorylates PIP to PIP2 on the inner leaflet of the cell membrane, which is subsequently used as a substrate by PI3K to generate PIP3 (Fig. 1C; [55]). Akt, a key protein in the Akt/PI3K signalling pathway, is a serine/threonine-specific protein kinase that plays a crucial role in the progression of several human cancers when phosphorylated by PIP3 [25, 56, 57]. In addition to the effect of Mak proteins on *C. elegans* PIP2 and PIP5K1α levels, we have recently shown that the MakA protein by itself can modulate mammalian host cell lipid biosynthesis in HCT8 cells, grown in vitro [58]. Moreover, we recently reported that phosphatidic acid (PA)-mediated MakA-binding to the plasma membranes of host cells promoted macropinocytosis, which led to the formation of an endomembrane-rich aggregate, resulting in the induction of autophagy and dysfunction of intracellular organelles. HCT8 cells were utilized as a model system to examine further how MakA protein influences PIP5K1α-dependent lipid signalling in human cells. HCT8 cells were treated with Alexa568MakA (500 nM, 48 h) and stained for PIP5K1α in order to determine if MakA co-localizes with PIP5K1α.

**Fig. 2:**
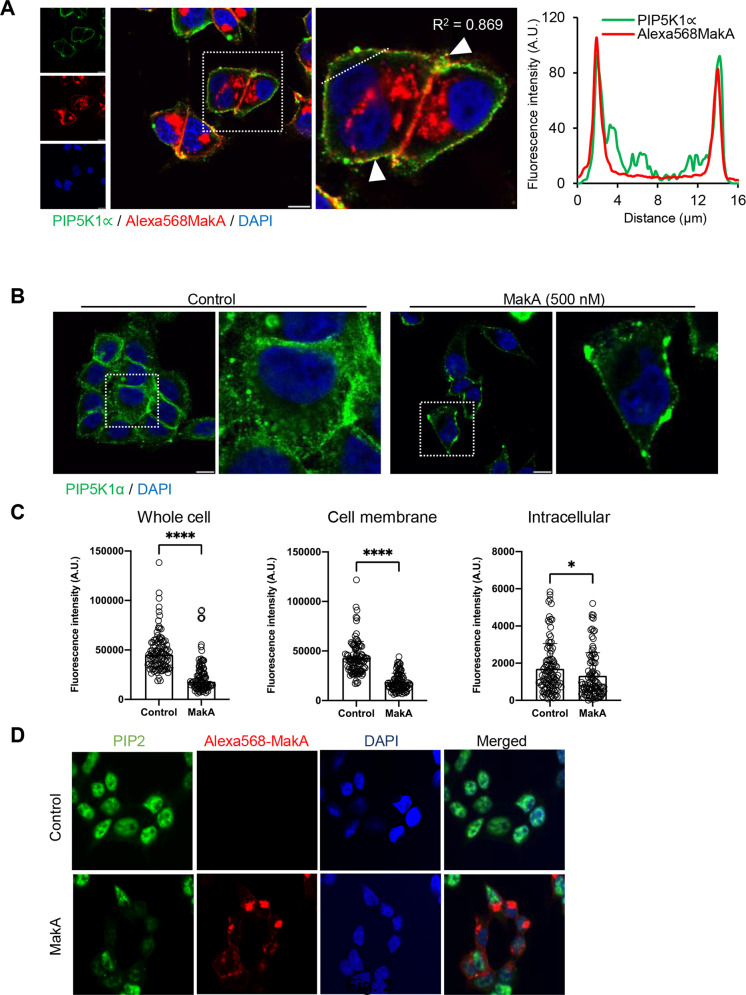
MakA and PIP5K1α interaction and reduction of PIP2 level in MakA treated HCT8 cells. **A** HCT8 colon carcinoma cells were treated with Alexa568MakA (500 nM, red) for 48 h and stained for PIP5K1α (green) using a specific antibody. Arrowhead (white) indicates cell membrane bound MakA. Nuclei were counterstained with DAPI (blue). Scale bars, 10 μm. Graph shows fluorescence intensity profiles of the corresponding image in along the dotted white line. Pearson correlation co-efficient was used for calculation of Alexa568MakA (red) co-localization with PIP5K1α (green) along the cell membrane. **B** HCT8 cells were treated with Alexa568MakA (500 nM) for 48 h and stained for PIP5K1α (green) using a specific antibody. Scale bars = 20 μm. **C** Histograms indicate quantification of cells (*n* = 90–100) presented from experiment in (**B**). *p ≤ 0.05 and ****p ≤ 0.0001. **D** Confocal microscopic analysis showing the levels of PIP2 in HCT8 cells treated with Alexa568MakA (red). PIP2 was stained using anti-PIP2 mouse antibody (green) and nuclei were counterstained with DAPI (blue). Scale bar = 20 μm.

**Fig. 3:**
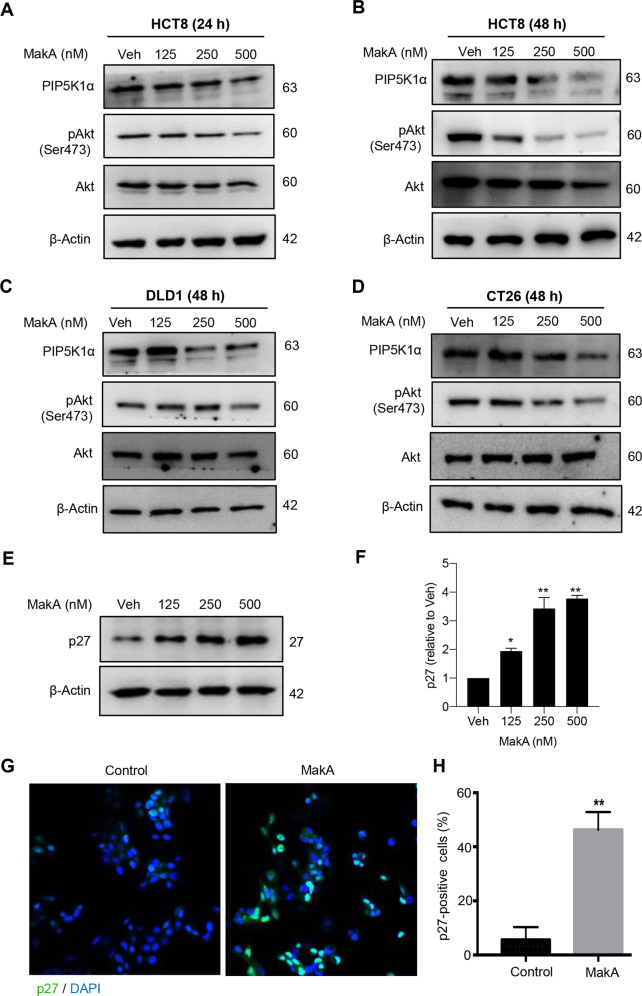
MakA inhibits PIP5K1α, and Akt activity, and induces p27 expression. **A**–**D** PIP5K1α, pAkt, and total Akt levels are shown in immunoblot images of HCT8, DLD1, and CT26 cancer cells treated with increasing concentrations of MakA, as indicated. The results are representative of at least two independent experiments. **E** Western blot analysis of HCT8 cells treated with increasing concentrations of MakA reveals the levels of p27 protein expression. **F** Histograms illustrate the quantification of p27 relative to total actin from the experiment in (**E**). Data are representative of three independent experiments and are expressed relative to cells treated with vehicle (20 mM Tris-HCl); bar graphs show mean ± s.d. The significance of replicates was determined using a one-way analysis of variance *p ≤ 0.05 and **p ≤ 0.01. **G** Representative confocal microscopy images of p27 expression (green) after treatment of HCT8 cells with or without MakA. Nuclei were counterstained with DAPI (blue). **H** A bar graph displaying the percentage of p27-positive cells in HCT8 cells treated with vehicle or MakA. Quantification is obtained from the experiment in (**G**).

Using confocal microscopy, we detected PIP5K1α co-localization with Alexa568MakA primarily at the cell membrane of intoxicated cells (Fig. 2A). Moreover, we observed a significant decrease in the level of PIP5K1α in the MakA-treated HCT8 cells (Fig. 2B, C). We observed a more significant decrease of PIP5K1α in the membrane of intoxicated cells than that of other cellular compartments (Fig. 2C). To determine if the reduction in fluorescence intensity of PIP5K1α in response to MakA affects its activity, specific antibodies were used to stain HCT8 cells for PIP2. HCT8 cells treated with 500 nM Alexa568MakA for 48 h had a reduced PIP2 level (Fig. 2D). Western blot analysis revealed a dose- and time-dependent decrease in PIP5K1α expression in HCT8 cells (Fig. 3A, B and Fig. S1A). In addition, we examined the level of PIP5K1α in two additional (human DLD1 cells and murine CT26 cells) colon cancer cell lines to investigate whether the effect of MakA would be similar in different cell lines. The immunoblot analysis revealed a dose-dependent decrease in PIP5K1α expression in DLD1 and CT26 cancer cells treated with MakA for 48 h, essentially similar to the effect found in HCT8 cells (Fig. 3C, D and Fig. S2A, B). Then, we anticipated that decreased levels of PIP5K1α and PIP2 in MakA-treated HCT8 cells could influence the expression of phospho-Akt, Ser 473 (pAkt), a crucial downstream signalling molecule in cancer cell survival and proliferation. By western blot analysis, we observed a time- and dose-dependent decrease in the phosphorylation of Akt when HCT8 cells were treated with MakA (Fig. 3A, B and Fig. S1A). Moreover, immunofluorescence microscopic analysis confirmed the reduced protein expression level of pAkt in MakA-treated HCT8 cells (Fig. S1B). Additionally, MakA treatment of DLD1 and CT26 cancer cells for 48 h also showed a dose-dependent decrease in the expression of pAkt (Fig. 3C, D and Fig. S2A, B). Taken together, our findings suggest that MakA may target the PI3K/Akt lipid signalling pathway in colon cancer cells by downregulating the expression of PIP5K1α and inhibiting Akt. Our results imply that the intoxication of tumour cells with MakA may result in the inhibition of PIP5K1α activity.

### MakA induces p27 expression, downregulates the expression of cell cycle regulatory proteins, and triggers G2/M cell cycle arrest in HCT8 cells

**Fig. 4:**
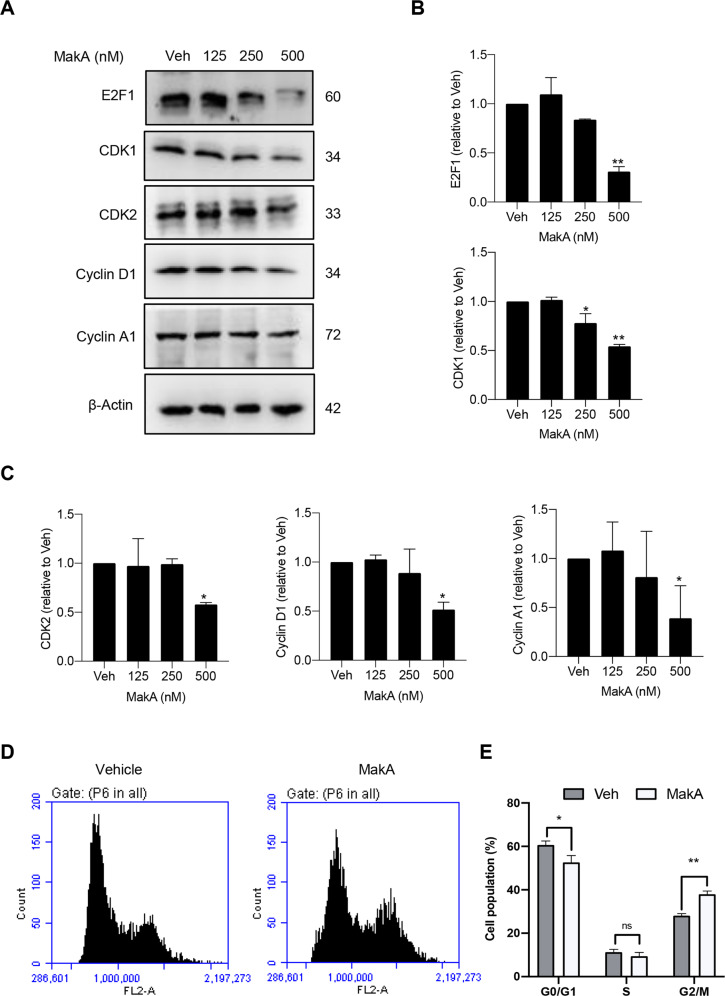
MakA induces G2/M cell cycle arrest in HCT8 cells and alters the expression of cell cycle regulatory proteins. **A** Western blot analysis shows levels of E2F1, CDK1, CDK2, cyclin D1, and cyclin A in HCT8 cells treated with increasing concentrations of MakA (24 h). **B**, **C** Histograms indicate the normalized quantification of E2F1, CDK1, CDK2, cyclin D1, and cyclin A1 relative to total actin. Data is representative of at least two independent experiments and is expressed compared to cells treated with vehicle (20 mM Tris-HCl); bar graphs show mean ± s.d. Significance was determined using a one-way analysis of variance (ANOVA) followed by Dunnett’s post-test in comparison to the vehicle control *p ≤ 0.05 and **p ≤ 0.01. **D** Cell cycle distribution flow cytometric analysis of HCT8 cells treated with MakA protein (500 nM, 24 h). **E** Histogram showing the distribution of HCT8 cells during each phase of the cell cycle after treatment with vehicle or MakA. Data are representative of three independent experiments. *p ≤ 0.05 and **p ≤ 0.01.

The protein p27, an inhibitor of cyclin-dependent kinase (CKI), is one of the key downstream targets of Akt. In cancer cells, Akt phosphorylates p27 at Thr-157, resulting in cytoplasmic localization and proteasomal destruction of p27 [59, 60]. This results in the downregulation of p27, rendering it unable to perform its nuclear function as a cell cycle inhibitor [61, 62]. Considering inactivation of Akt in cells treated with MakA protein, we anticipated that MakA treatment of HCT8 cells may result in increased p27 levels and hence inhibition of cell cycle progression. To determine the expression levels of p27 and of cell cycle regulatory proteins such as E2F1, cyclin-dependent kinase 1 and 2 (CDK1 and 2), cyclin D1, and cyclin A1, we performed western blot analyses on samples from MakA-treated cells. Upon MakA treatment, the cells indeed exhibited a concentration-dependent increase in p27 levels (Fig. 3E, F). The increase in p27 expression was further confirmed by confocal microscopy, which revealed a higher number of p27-positive HCT8 cells after treatment with the MakA protein (Fig. 3G, H). Moreover, western blot analyses demonstrated dose-dependent decreases in cellular levels of all tested cell cycle proteins (E2F1, CDK1, CDK2, cyclin D1, and cyclin A1) as a result of MakA treatment (Fig. 4A–C). We also observed a dose-dependent decrease in the levels of cyclin D1 in MakA-treated DLD1 and CT26 colon cancer cells (Fig. S3A, B). Using flow cytometry, we analyzed the MakA-mediated effects on cell cycle progression. Compared to vehicle-treated cells, MakA treatment led to a significant decrease in the proportion of cells in the G0/G1 phase but had no impact on the S phase cell population (Fig. 4D, E). Notably, MakA increased the proportion of HCT8 cells in the G2/M phase (*p* ≤ 0.01) (Fig. 4D, E), indicating that MakA promotes G2/M cell cycle arrest in HCT8 cells and inhibits their entrance into the G0/G1 phase. Taken together, our results suggest that MakA causes G2/M cell cycle arrest in vitro by altering the levels of several cell cycle regulators.

### MakA inhibits PIP5K1α-dependent tumour cell proliferation

**Fig. 5:**
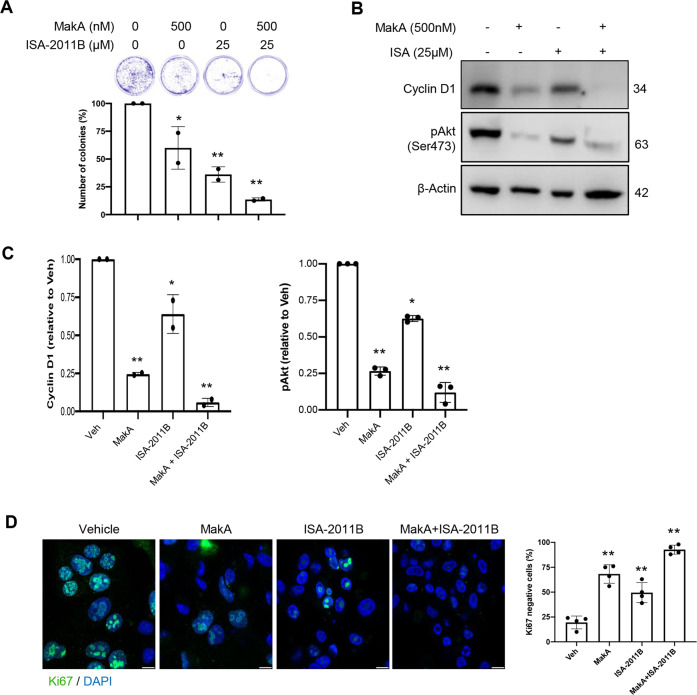
MakA inhibits tumour cell proliferation in a PIP5K1α-dependent manner. **A** MakA and PIP5K1α inhibitor, ISA-2011B synergistically inhibited HCT8 cell proliferation as quantified by colony forming assay. Data are representative of two independent experiments; bar graphs show mean ± s.d. Significance from replicates was determined using a one-way analysis of variance (ANOVA) with Dunnett’s multiple comparisons test (post-test) against vehicle (Veh). *p ≤ 0.05 and **p ≤ 0.01. **B** Western blot analyses of cyclin D1 and pAkt levels in HCT8 cells upon 48 h treatment with MakA (500 nM), ISA-2011B (25 μM) or a combination of MakA (500 nM) and ISA-2011B (25 μM). β-actin was used as a loading control. **C** Histograms showing the normalized quantification of cyclin D1 and pAkt relative to total actin. Data are representative of at least two independent experiments; bar graphs show mean ± s.d. Significance from replicates was determined using a one-way analysis of variance (ANOVA) with Dunnett’s multiple comparisons test (post-test) against vehicle (Veh). *p ≤ 0.05 and **p ≤ 0.01. **D** Confocal microscopy analysis of vehicle (Veh), MakA (500 nM), ISA-2011B (25 μM), or a combination of MakA (500 nM) and ISA-2011B (25 μM) treated HCT8 cells (48 h treatment). Cells were stained with cell proliferation marker, Ki67 (green). Nuclei were counterstained with DAPI (blue). Inhibition of HCT8 cell proliferation was quantified by an increase in the number of Ki67-negative stained cells from four individual microscopy fields of view (65-165 cells per field of view). *p ≤ 0.05 and **p ≤ 0.01. Scale bar = 20 μm.

We recently reported that MakA inhibits the proliferation of tumour cells [5]. To investigate if PIP5K1α plays a role in MakA-dependent suppression of tumour cell proliferation, HCT8 cells were treated with (i) MakA, (ii) PIP5K1α inhibitor, ISA-2011B [63], or (iii) a combination of MakA and ISA-2011B, and cell proliferation was quantified using a Clonogenic assay. Consistent with our previous results, we observed a reduction in proliferation of HCT8 cells treated with MakA (Fig. 5A). Similarly, ISA-2011B showed a significant reduction in tumour cell proliferation (Fig. 5A). Notably, the combination of MakA and ISA-2011B inhibited cell proliferation more strongly (Fig. 5A). To determine if the combination of MakA and ISA-2011B induces stable inhibition of tumour cell proliferation, HCT8 cells were treated with (i) MakA, (ii) ISA-2011B, or (iii) a combination of MakA and ISA-2011B and stained for cell proliferation markers, cyclin D1 or Ki67 (Fig. 5B–D). When HCT8 cells were exposed to MakA or ISA-2011B alone, we observed a decrease in expression of cyclin D1 and an increase in the number of Ki67-negative cells (Fig. 5B–D). However, when a combination of MakA and ISA-2011B was applied, the effect was considerably more pronounced (Fig. 5B–D). HCT8 cells were treated with (i) MakA, (ii) ISA-2011B, or (iii) a combination of MakA and ISA-2011B to determine whether the combination of MakA and ISA-2011B would affect Akt signalling per se. Immunoblot analysis revealed a reduction in pAkt expression in HCT8 cells treated with either MakA or ISA-2011B alone (Fig. 5B, C). Similarly, to the effect on the cyclin D1 levels, the combined treatment with MakA and ISA-2011B caused a more pronounced decrease in pAkt protein level than treatment with either inhibitor (Fig. 5B, C).

**Fig. 6:**
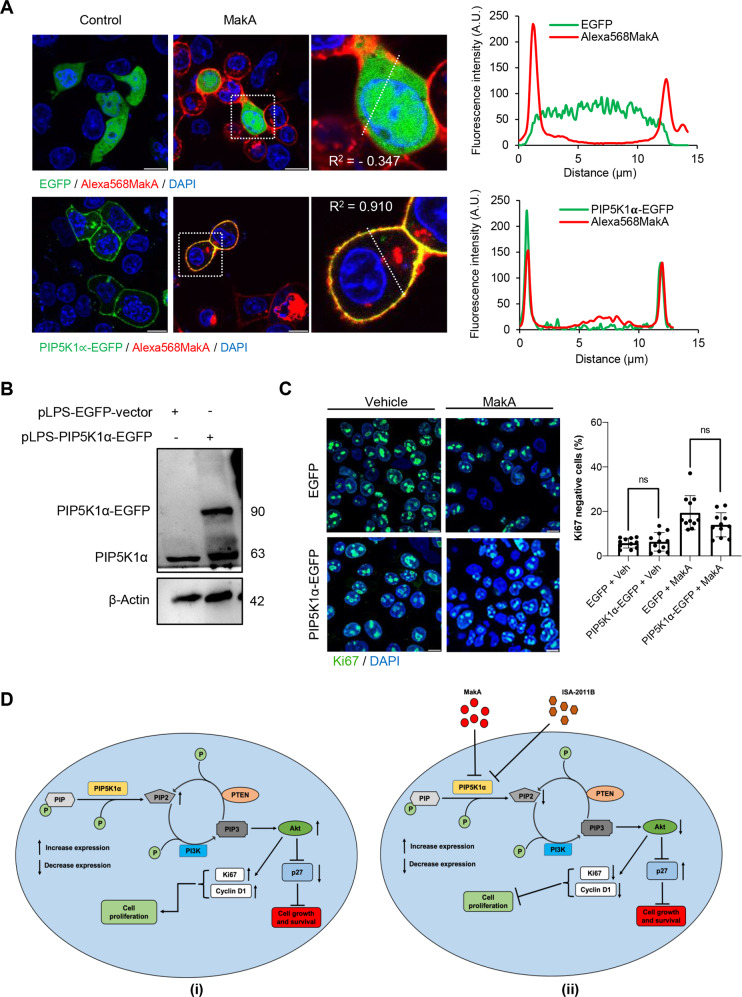
Effect of MakA in HCT8 cells with PIP5K1α overexpression and schematic summaries of the MakA effects on PIP5K1α-mediated signalling. **A** HCT8 cells were transfected with EGFP-vector control (upper panel) or PIP5K1α-EGFP (lower panel) plasmids as described in “Materials and methods”. For co-localization experiments of MakA and PIP5K1α, the transfected HCT8 cells were subsequently treated with Alexa568MakA (250 nM, red) for 48 h. Nuclei were counterstained with DAPI (blue). Graphs on the right show fluorescence intensity profiles of the corresponding images along the dotted white lines. Pearson correlation co-efficient was used for calculation of Alexa568MakA (red) co-localization with EGFP or PIP5K1α-EGFP (green) along the cell membrane. Scale bars = 10 μm. **B** Western blot analysis of PIP5K1α levels in EGFP-vector control or PIP5K1α-EGFP HCT8 total cell lysates. **C** Confocal microscopy analysis of vehicle (Veh) or MakA (250 nM) treated (48 h treatment) HCT8 cells transfected with EGFP-vector control or PIP5K1α-EGFP plasmids. Cells were stained with cell proliferation marker, Ki67 (green). Nuclei were counterstained with DAPI (blue). Effects on HCT8 cell proliferation was assessed by quantifying the number of Ki67-negative cells from eleven individual microscopy fields of view (65–200 cells per field of view). Significance was determined using an unpaired t test, ns non-significant. **D** Schematic representations showing (i) the downstream signalling of PIP5K1α during tumour cell proliferation and survival, (ii) the effect of MakA and ISA-2011B inhibition.

In addition, we examined whether the overexpression of PIP5K1α would have a rescue effect on the reduced cell proliferation caused by MakA. First, we investigated the localization of MakA in HCT8 cells harbouring vector control plasmid (EGFP) or PIP5K1α over expressing plasmid. We observed a distinct co-localization of MakA and PIP5K1α primarily on the plasma membrane of HCT8 cells (Fig. 6A). By western blot analysis, we confirmed the overexpression of PIP5K1α in HCT8 transfected cells (Fig. 6B). HCT8 cells were stained with the cell proliferation marker Ki67 to determine if the overexpression of PIP5K1α might restore the proliferation of MakA-intoxicated cells. As shown in Fig. 6C, the number of Ki67-negative cells appeared somewhat decreased in cells overexpressing PIP5K1α in comparison with that of the EGFP-vector control cells. However, the difference was not statistically significant (*p* = 0.0712), and overexpression of PIP5K1α in HCT8 cells appeared to cause no or little rescue effect towards the MakA. Together, the data suggest that MakA caused efficient inhibition of tumour cell proliferation in a PIP5K1α-interacting manner (Fig. 6D).

## Discussion

In this present study, we used *C. elegans*, HCT8, DLD1, and CT26 cancer cells as our models to investigate the mechanisms of the effect of MakA on the host PIP5K1α/PI3K/Akt phospholipid signalling. By immunoblot analyses and immunofluorescence imaging, we found that MakA caused reduced expression levels of PIP5K1α and PIP2 in both *C. elegans* and in different mammalian cancer cells.

The phosphatidylinositol 4,5-bisphosphate (PIP2) has emerged as an important regulator of many ion channels and transporters [64]. In some cases, the regulation is known to involve specific lipid-protein interactions, but the mechanisms by which PIP2 regulates many of its various targets remain to be fully elucidated. Moreover, in *C. elegans*, PIP2 plays an important role in generation of rhythmic inositol 1,4,5-trisphosphate (IP3)-dependent Ca^++^ oscillations that control muscle contractions required for defecation [65]. In our previous studies [4] we found that worms fed with *E. coli* Top10/pBAD18 exhibited a typical defecation rhythm, which included regular contractions of posterior body-wall muscles and regular expulsions during normal defecation periods. On the other hand, defecation was severely impaired in worms fed with *E. coli* expressing Mak proteins [4]. In the light of the present findings, it seems plausible that the defecation impairment observed in our earlier studies with the worms may be due to the effect of MakA on PIP2 levels.

PIP5K1α has been directly linked in both breast cancer and prostate cancer (PCa), and researchers have shown that greater levels correspond with worse patient outcomes [28, 66]. Recent research has shed light on the potential therapeutic usefulness of targeting PIP5K1α in the treatment of malignancies, in addition to its primary role in the PI3K–Akt signalling cascade. An earlier study led to the discovery of a selective kinase inhibitor of PIP5K1α as a new anticancer drug candidate known as ISA-2011B. This small molecule compound was shown to successfully suppress the development of PCa tumours in vivo as well as the invasion of PCa cells in vitro [63]. The PIP5K1α-associated PI3K/Akt and the downstream proliferation, survival, and invasion pathways were all targeted by ISA-2011B, which was demonstrated to supress tumour growth and metastasis to distant organs in xenografted mice. In addition, very recently, the powerful inhibition of PIP5K1α as well as a number of other kinases was identified in studies with a molecule known as GSK2291363, which is an indazole-pyrimidine with a restricted spectrum [67].

A few studies investigated the interaction between bacterial toxins and lipids, in particular the direct interaction with PIP2. These toxins include *Vibrio parahemolyticus* VopR toxin, *Pseudomonas fluorescens* ExoU toxin, *Yersinia* YopO toxin, *Salmonella* SopF toxin, and *Bordetella* T3SS effector protein BteA [68, 69]. However, the effects of these toxins on lipid kinase-mediated signalling have not been investigated in detail. In addition, *Pasteurella multocida* PMT toxin is also known as cyclomodulin, a protein that promotes or interferes with the progression of the cell cycle in target cells by upregulating multiple signalling cascades. These signalling cascades include Rho, phospholipase C, and mitogen-activated protein kinases (MAPKs) such as c-Jun N-terminal kinase (JNK) and extracellular signal-regulated kinase (ER) [70]. However, as of yet, there is little evidence to suggest that bacterial toxins interact with lipid kinases, specifically PIP5K1α, in order to alter the course of the cell cycle.

In our studies, we discovered that treating HCT8 cells with MakA resulted in a substantial reduction in the amount of PIP5K1α protein level, as well as a colocalization of membrane bound MakA with PIP5K1α. In addition to this, MakA was shown to decrease the levels of Akt and phosphorylated Akt (ser-473), while simultaneously elevating the expression of the cyclin-dependent kinase inhibitor p27. This increase in p27 was verified by confocal microscopy, which showed that there were more p27-positive HCT8 cells after being treated with MakA. In HCT8 cells treated with MakA, an increased amount of p27 affected downstream signalling targets among cell cycle regulatory proteins (E2F1, CDK1, CDK2, cyclin D1, and cyclin A1). Flow cytometry analysis of the cell cycle revealed that MakA affected cell cycle distribution. There was a decrease in the percentage of cells in the G1 phase and an increase in the proportion of cells in the G2/M phase, which led to an arrest in the G2/M phase of the cell cycle.

Many studies are now being conducted to determine the efficacy of combination treatments, combining several anticancer agents to overcome the limits of currently available anticancer therapies [71]. Such combination therapies are also anticipated to have synergistic anticancer effects. In this study, we investigated the effect of MakA on PIP5K1α/Akt signalling and also its synergistic interaction with ISA-2011B. The combination of MakA and ISA-2011B considerably enhanced the suppression of tumour cell proliferation. We anticipate that our research will raise interest in the potential anticancer effects of MakA and ISA-2011B, especially their synergistic interactions. However, the molecular mechanisms underlying this synergistic effect require additional studies for clarification and, for example, it will be of interest to investigate whether there is some direct interaction between MakA and PIP5K1α. Nevertheless, to the best of our knowledge, MakA is the first bacterial protein shown to target the PIP5K1α lipid-signalling pathway in host cells.

## Supplementary information

Reproducibility checklist

Supplementary figure legends

Supplementary Figure 1

Supplementary Figure 2

Supplementary Figure 3

## Data Availability

All data sets generated and analyzed during this study are included in the article and its supplementary Information files. Additional data are available from the corresponding author on reasonable request.
