# Supplementary material for: Bacterial protein MakA causes suppression of tumour cell proliferation via inhibition of PIP5K1α/Akt signalling
Source: Cell Death Dis. 2022 Dec 6;13(12):1024. doi: 10.1038/s41419-022-05480-7 (PMC9726977; doi:10.1038/s41419-022-05480-7)
Supplement: Supplementary file 2 — Supplementary figure legends [file 41419_2022_5480_MOESM2_ESM.docx]

**Supplementary information**

**Bacterial protein MakA causes suppression of tumour cell proliferation via inhibition of PIP5K1α/Akt signalling**

**Eric Toh^1,2^, Palwasha Baryalai^1,2^, Aftab Nadeem^1,2^, Kyaw Min Aung^1,2^ Sa Chen^1^, Karina Persson^3^, Jenny L Persson^1^, Bernt Eric Uhlin^1^, Sun Nyunt Wai^1,2 *^**

^1^ Department of Molecular Biology, Umeå Centre for Microbial Research (UCMR), Umeå University, SE-90187 Umeå, Sweden.

^2^ The Laboratory for Molecular Infection Medicine Sweden (MIMS),

Umeå University, SE-90187 Umeå, Sweden,

^3^ Department of Chemistry, Umeå University, SE-90187 Umeå, Sweden,

* Corresponding author: Sun Nyunt Wai

E-mail: sun.nyunt.wai@umu.se

ORCID: 0000-0003-4793-4671

**Supplementary figure legends**

**Fig. S1: MakA inhibits Akt phosphorylation in HCT8 tumour cells.**

**(A)** Histograms show quantification of Western blot analysis for PIP5K1α and pAkt (Ser473) normalized against β-actin or pAkt (Ser473) data normalized against β-actin and total Akt. Data are representative of two or three independent experiments; bar graphs show mean ± s.d. Significance from replicates was determined using a one-way analysis of variance (ANOVA) with Dunnett's multiple comparisons test (post-test) against vehicle (Veh). *p ≤ 0.05 and ** p ≤ 0.01. **(B)** Confocal microscopy analysis images showing expression of pAkt (green) in control or MakA-treated HCT8 cells (48 h, 500 nM). Nuclei were counterstained with DAPI (blue). Scale bar = 20 μm.

**Fig. S2:** **MakA inhibits Akt phosphorylation in DLD1 and CT26 tumour cells.**

**(A-B)** Histograms show quantification of Western blot analysis for PIP5K1α and pAkt (Ser473) normalized against β-actin or pAkt (Ser473) data normalized against β-actin and total Akt in MakA-treated DLD1 and CT26 cancer cells respectively. Data are representative of atleast two independent experiments; bar graphs show mean ± s.d. Significance from replicates was determined using a one-way analysis of variance (ANOVA) with Dunnett's multiple comparisons test (post-test) against vehicle (Veh). *p ≤ 0.05 and ** p ≤ 0.01.

**Fig. S3:** **MakA inhibits cyclin D1 expression in DLD1 and CT26 tumour cells**

**(A-B)** Western blot analysis shows levels of cyclin D1 in MakA-treated (48 h) DLD1 and CT26 colon cancer cells respectively. Histograms to the right indicate the normalized quantification of cyclin D1 relative to β-actin. Data is representative of two independent experiments and is expressed compared to cells treated with vehicle (20 mM Tris-HCl); bar graphs show mean ± s.d. Significance was determined using a one-way analysis of variance (ANOVA) followed by Dunnett's post-test in comparison to the vehicle control *p ≤ 0.05 and ** p ≤ 0.01.
