# Supplementary figures and images for: Bacterial protein MakA causes suppression of tumour cell proliferation via inhibition of PIP5K1α/Akt signalling
Source: Cell Death Dis. 2022 Dec 6;13(12):1024. doi: 10.1038/s41419-022-05480-7 (PMC9726977; doi:10.1038/s41419-022-05480-7)

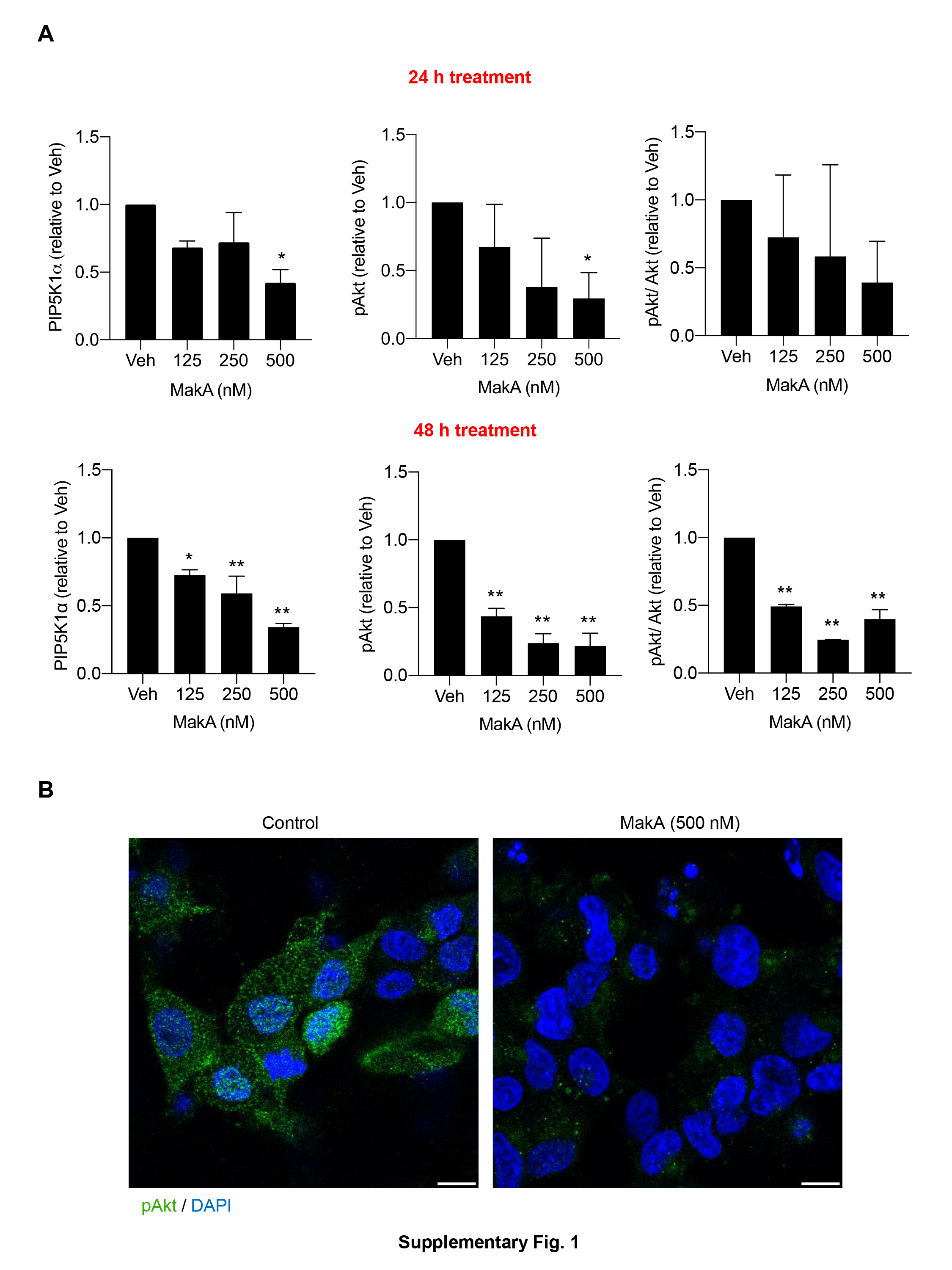

Supplement: Supplementary file 3 — Supplementary Figure 1 [file 41419_2022_5480_MOESM3_ESM.tif]

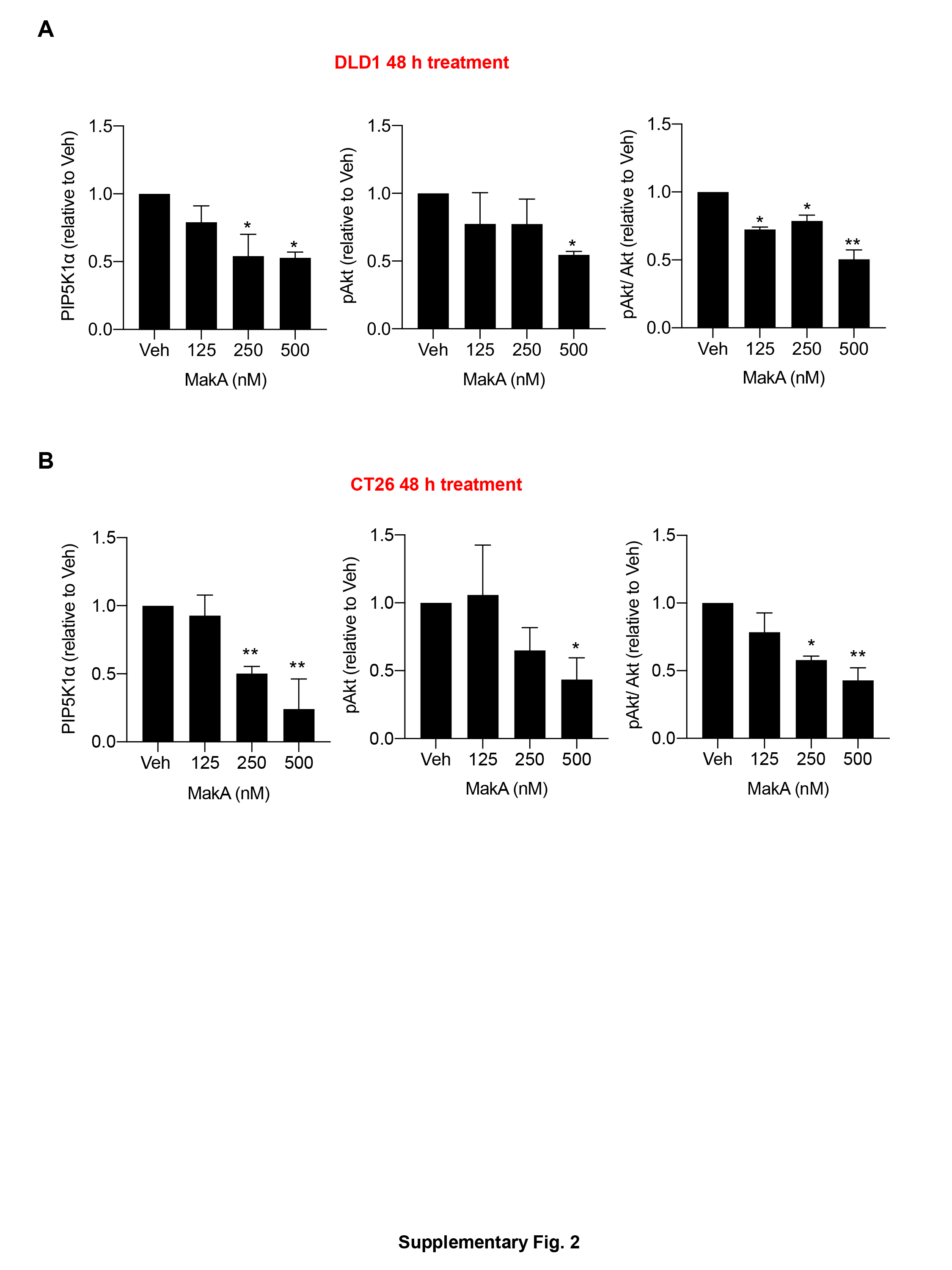

Supplement: Supplementary file 4 — Supplementary Figure 2 [file 41419_2022_5480_MOESM4_ESM.tif]

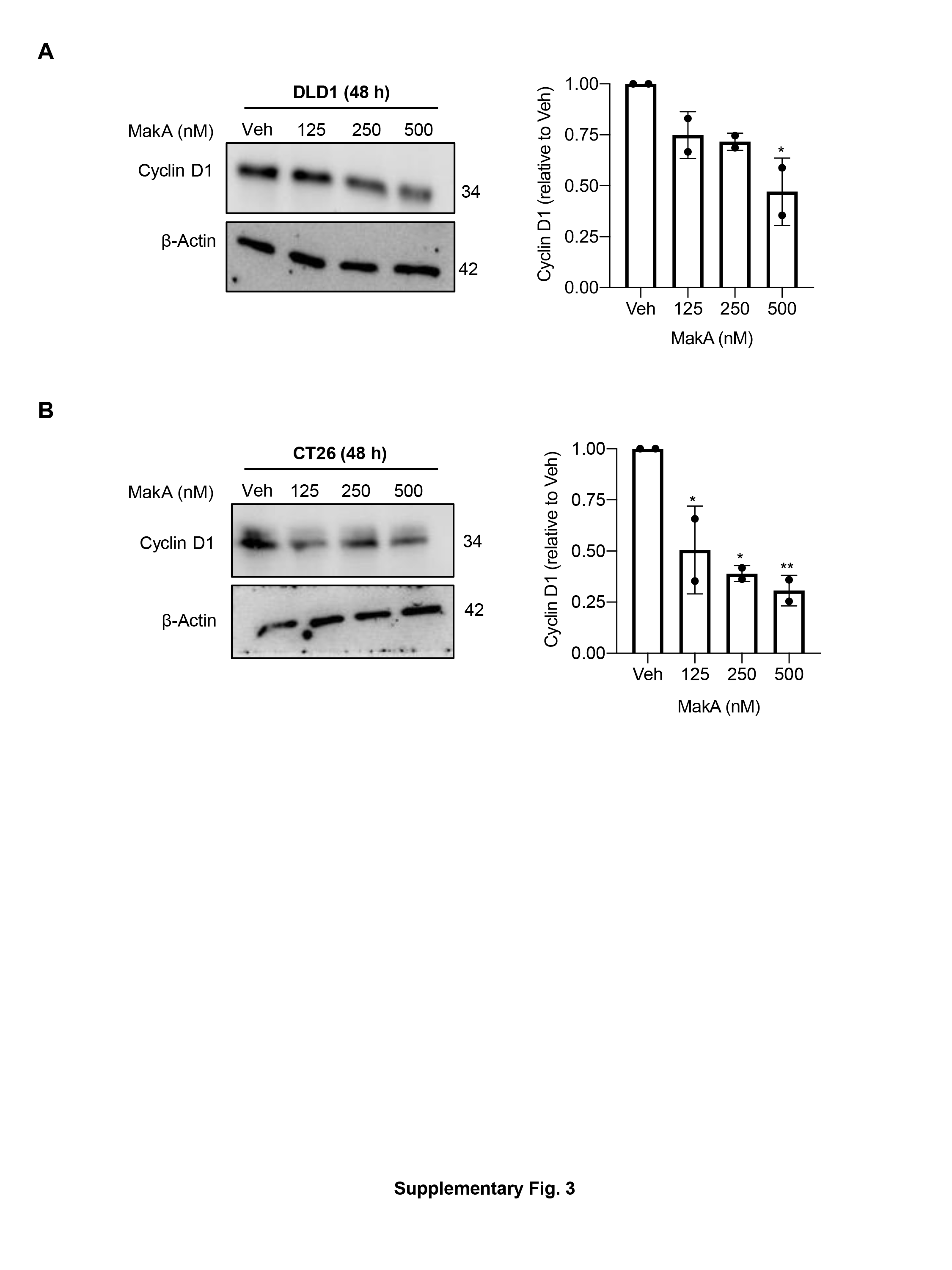

Supplement: Supplementary file 5 — Supplementary Figure 3 [file 41419_2022_5480_MOESM5_ESM.tif]
